# The fourth crystallographic closest packing unveiled in the gold nanocluster crystal

**DOI:** 10.1038/ncomms14739

**Published:** 2017-03-24

**Authors:** Zibao Gan, Jishi Chen, Juan Wang, Chengming Wang, Man-Bo Li, Chuanhao Yao, Shengli Zhuang, An Xu, Lingling Li, Zhikun Wu

**Affiliations:** 1 Key Laboratory of Materials Physics, Anhui Key Laboratory of Nanomaterials and Nanotechnology, CAS Center for Excellence in Nanoscience, Institute of Solid State Physics, Chinese Academy of Sciences, Hefei 230031, China; 2 Key Laboratory of Ion Beam Bioengineering, Institute of Technical Biology and Agriculture Engineering, Chinese Academy of Sciences, Hefei 230031, China; 3 Hefei National Laboratory for Physical Sciences at the Microscale, University of Science and Technology of China, Hefei 230026, China; 4 Instrumental Analysis Center, Shanghai Jiaotong University, Shanghai 200240, China

## Abstract

Metal nanoclusters have recently attracted extensive interest not only for fundamental scientific research, but also for practical applications. For fundamental scientific research, it is of major importance to explore the internal structure and crystallographic arrangement. Herein, we synthesize a gold nanocluster whose composition is determined to be Au_60_S_6_(SCH_2_Ph)_36_ by using electrospray ionization mass spectrometry and single crystal X-ray crystallography (SCXC). SCXC also reveals that Au_60_S_6_(SCH_2_Ph)_36_ consists of a fcc-like Au_20_ kernel protected by a pair of giant Au_20_S_3_(SCH_2_Ph)_18_ staple motifs, which contain 6 tetrahedral-coordinate *μ*_4_-S atoms not previously reported in the Au–S interface. Importantly, the fourth crystallographic closest-packed pattern, termed 6H left-handed helical (6HLH) arrangement, which results in the distinct loss of solid photoluminescence of amorphous Au_60_S_6_(SCH_2_Ph)_36_, is found in the crystals of Au_60_S_6_(SCH_2_Ph)_36_. The solvent-polarity-dependent solution photoluminescence is also demonstrated. Overall, this work provides important insights about the structure, Au–S bonding and solid photoluminescence of gold nanoclusters.

## Abstract (web summary)

Metal nanoclusters are explored for their precise structures and compelling properties. Here, the authors synthesize a gold cluster with unique structural features, including giant staple motifs, tetrahedral-coordinate *μ*_4_-S atoms, and a helical closest-packed crystallographic pattern that influences the cluster's photoluminescence.

Bridging gold atom (or gold complexes)^1^,^2^,^3^ and nanocrystals (typically >3 nm)^4^,^5^,^6^,^7^,^8^, gold nanoclusters^9^,^10^,^11^,^12^,^13^,^14^,^15^ have recently attracted increasing research interest due to their well-defined compositions and structures, unique properties and potential applications. In particular, the structures of gold nanoclusters are of primary significance and have received the most extensive attention in the research community^16^,^17^,^18^,^19^,^20^,^21^. However, structure achievements remain limited due to the difficulty of precise size control and structural resolution, especially for relatively large gold nanoclusters^22^,^23^,^24^,^25^,^26^. It is generally believed that the gold nanoclusters adopt kernel-staple structures, and the staple motifs play a vital role in stabilizing the nanoclusters. The large staple motif Au_8_(SR)_8_ was reported in Au_20_(SR)_16_ nanocluster^27^, but the question remains whether there is a larger staple motif. Although thiolate or sulfido (-S-) can be found in the surface of gold nanoclusters as protecting ligands, both thiolate sulfur and sulfido (-S-) are always three-coordinate (mainly *μ*_2_-S and rarely *μ*_3_-S)^28^,^29^, naturally raising the question of whether there are sulfurs with any other coordination numbers in gold nanoclusters. Notably, four-coordinate *μ*_4_-S and five-coordinate *μ*_4_-S have been reported in Cr(III) and Cu(I) complexes^30^,^31^, respectively, but such high-coordinate sulfur has not been found in Au–S interfaces (not limited to gold nanoclusters).

In addition to these internal structure questions, there are some other crystallographic arrangement issues that need to be addressed. It is known that there are two classic crystallographic closest packings for atomic crystals: the fcc arrangement with a packing sequence of ‘ABC' found in bulk Au, Ag, Pd, Pt and Ir metals^32^, FePt nanoparticles^33^, and others; and the hcp or 2H arrangement with a packing sequence of ‘AB' revealed in Rh nanosheets^34^, Au square sheets^35^, PbPt nanorods^36^, BiPt nanoplates^37^, among others. The third closest packing, named the 4H arrangement, has a packing sequence of ‘ABCD' and was first observed in bulk Ag by Novgorodova *et al*. in 1979 (ref. 38), then subsequently revealed in Ag nanocrystals and nanowires^39^,^40^, Au nanoribbons^41^ and more. The advances in gold nanocluster research provide opportunities to discover novel closest-packed patterns. It is worth noting that in contrast to gold nanocrystals, molecule-like gold nanoclusters can grow high-quality single crystals for X-ray crystallography analyses. Thus, not only the internal structure (atom packing in every gold nanocluster) but also the crystallographic arrangement (the arrangement of gold nanoclusters in single crystals) of gold nanoclusters can be resolved by single crystal X-ray crystallography (SCXC). Jin *et al*. reported the fcc crystallographic arrangement of Au_30_(SR)_18_ nanoclusters in early 2016 (ref. 42), and very recently, Wu *et al*. reported the 4H crystallographic arrangement in Au_92_ crystals^43^. It is currently unknown whether there are any other intriguing arrangement patterns in gold nanoclusters. To address this question, together with the above mentioned issues, more gold nanoclusters with complex surfaces must be synthesized, and their structures must be resolved.

Herein, we report the synthesis, structure (including internal and crystallographic) and photoluminescence of a gold nanocluster whose composition is determined to be Au_60_S_6_(SCH_2_Ph)_36_ using electrospray ionization mass spectrometry (ESI-MS) and SCXC. In particular, we find a fourth closest packing in the crystal of Au_60_S_6_(SCH_2_Ph)_36_.

## Results

### Synthesis and characterization

**Figure 1:**
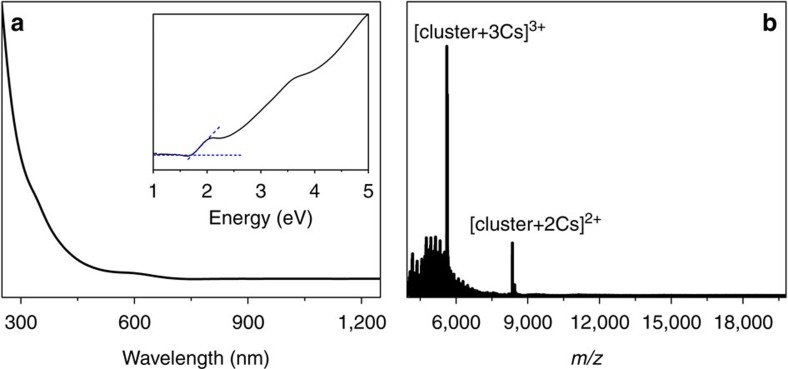
Characterization of Au_60_S_6_(SCH_2_Ph)_36_. Ultraviolet-visible-near-infrared spectrum (**a**) and electrospray ionization mass spectrometry (ESI-MS) (positive ion mode) spectrum (**b**) of Au_60_S_6_(SCH_2_Ph)_36_. The inset of **a** represents the spectrum on the energy scale.

The Au_60_S_6_(SCH_2_Ph)_36_ nanocluster was obtained via a thermal-induced ligand exchange reaction of molecularly pure Au_38_(SC_2_H_4_Ph)_24_ with excess phenylmethanethiol (HSCH_2_Ph). Of note, the interesting thing in this synthesis is that the protecting ligand of the starting nanoclusters and the incoming ligand only have subtle difference in composition (-CH_2_-, see Supplementary Fig. 1), which may have some implications to other nanoparticles (including quantum dots) synthesis. Briefly, the reaction was initialized by dissolving 10 mg of Au_38_(SC_2_H_4_Ph)_24_ nanoclusters in 1 ml of toluene containing 0.5 ml of HSCH_2_Ph, with stirring. After proceeding overnight at 100 °C under nitrogen atmosphere, the reaction was terminated by the addition of plenty of methanol. The crude product was thoroughly washed with petroleum ether and methanol for four times, then subjected to subsequent separation and purification by preparative thin-layer chromatography (PTLC)^19^. Single crystals of the purified nanoclusters were grown by the vapour diffusion of acetonitrile into the toluene solution of the purified nanoclusters at 5 °C. Black-coloured crystals were formed after one week. As shown in Fig. 1a, the optical absorption spectrum of the as-obtained nanocluster has no dominant visible absorption peak and only shows a very weak absorption at∼345 nm (3.59 eV) and a step at∼600 nm (2.07 eV). The optical energy gap was determined to be ∼1.73 eV by extrapolating the lowest-energy absorption peak to zero absorbance (see the inset in Fig. 1a). The composition of the as-obtained nanocluster was identified by ESI-MS. Of note, without the addition of cesium acetate (CsOAc), no signal was observed in either positive or negative mode, which implies the charge neutrality of the nanocluster. To impart charges, CsOAc was added to the nanocluster solution to form positively charged [cluster+*x*Cs]^*x*+^ adducts in the electrospray process. As shown in Fig. 1b, two intense peaks at mass/charge ratio (*m/z*) 8,356.22 and 5,615.49 are observed, which can be readily assigned to [Au_60_S_6_(SCH_2_Ph)_36_Cs_2_]^2+^ (calculated: 8,356.31, deviation: 0.09) and [Au_60_S_6_(SCH_2_Ph)_36_Cs_3_]^3+^(calculated: 5,615.51, deviation: 0.02), respectively. Thus, the as-obtained nanocluster should be Au_60_S_6_(SCH_2_Ph)_36_, which was further confirmed by the subsequent SCXC analysis.

### Internal structure

**Figure 2:**
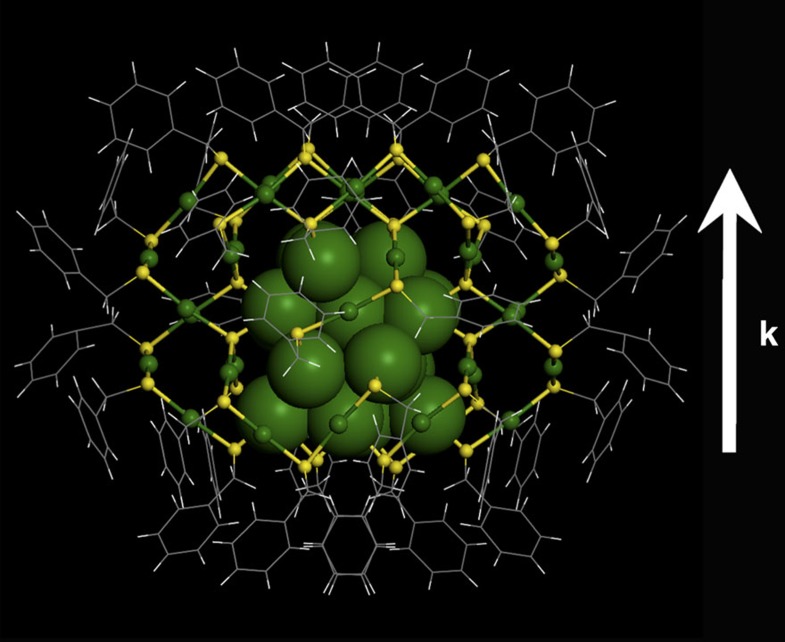
X-ray atomic structure of the Au_60_S_6_(SCH_2_Ph)_36_ nanocluster. The Au atoms are in green, the sulfur atoms are in yellow, and the benzyls are in wireframe. The vertical upward arrow indicates the direction of the **k**-vector.

**Figure 3:**
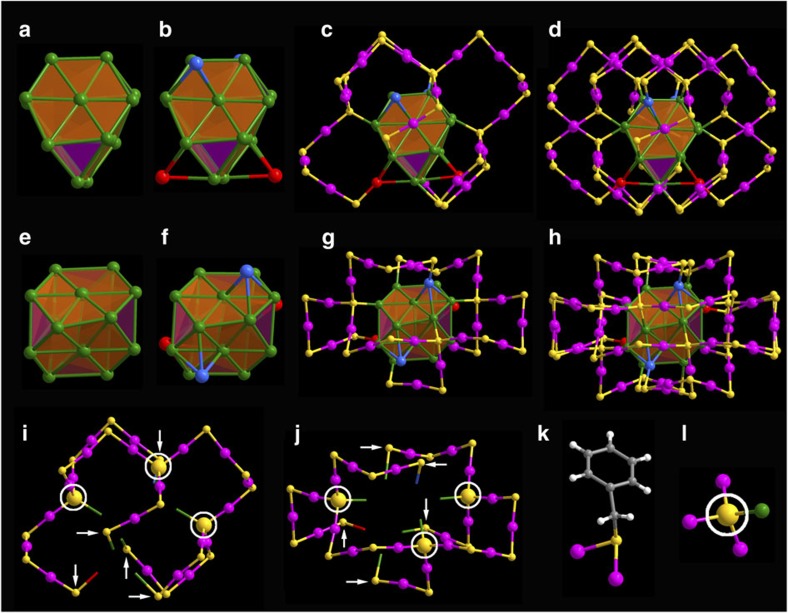
Anatomy of the atomic structure of Au_60_S_6_(SCH_2_Ph)_36_ nanocluster. (**a**,**e**) Side and top view of the Au_16_ tetrahedron without vertex; (**b**,**f**) side and top view of Au_20_ inner kernel; (**c**,**g**) side and top view of Au_20_ kernel with an Au_20_S_3_(SCH_2_Ph)_18_ staple motif; (**d**,**h**) side and top view of the overall framework of the Au_60_S_6_(SCH_2_Ph)_36_ nanocluster with a pair of Au_20_S_3_(SCH_2_Ph)_18_ staple motifs; (**i**,**j**) side and top view of the Au_20_S_3_(SCH_2_Ph)_18_ staple motif; (**k**) the *μ*_2_-S atom and (**l**) the *μ*_4_-S atom (highlighted in the white circle). Colour labels: yellow=S, white=H, gray=C, other colour=Au. The Au atoms are represented in different colours to differentiate from them each other on the bonding ways and occupying positions.

The structure of the as-obtained nanocluster was determined by SCXC. Figure 2 presents the total structure of the as-obtained nanocluster, which crystallizes in a hexagonal *P*6522 space group, has no centre or plane of symmetry and only possesses a *C*_2_ rotation axis of symmetry. The as-obtained nanocluster consists of an Au_20_ kernel and a pair of giant Au_20_S_3_(SCH_2_Ph)_18_ staple motifs. The Au_20_ kernel can be viewed as a fragment of the fcc structure in bulk gold or nanoparticles, typically >3 nm (diameter). The 16 gold atoms in the kernel constitute an Au_16_ tetrahedron without vertex (Fig. 3a,e), and each facet of the tetrahedron is a distorted hexagon. The other 4 Au atoms (highlighted in blue and red, Fig. 3b,f) in the kernel are capped on the four facets of the tetrahedron in a one-to-one fashion. Moreover, the Au_20_ kernel is protected by a pair of giant unanimous Au_20_S_3_(SCH_2_Ph)_18_ staple motifs: one Au_20_S_3_(SCH_2_Ph)_18_ staple connects to the Au_20_ kernel (Fig. 3c,g; the back view can be found in Supplementary Fig. 2) by five terminal *μ*_2_-S atoms (binding to one kernel Au atom, one staple Au atom and one -CH_2_Ph group, indicated by white arrow, see Fig. 3i,j) and three bridging *μ*_4_-S atoms (binding to one kernel Au atom and three staple Au atoms, highlighted in the white circle, see Fig. 3i,j), and the other Au_20_S_3_(SCH_2_Ph)_18_ staple binds to the Au_20_ kernel in the same fashion after rotating (180°) along the C_2_ axis of symmetry (Fig. 3d,h). It is worth noting that in addition to the common three-coordinate *μ*_2_-S atoms (binding to one R group and two Au atoms (Au–SR–Au), see Fig. 3k), there are six tetrahedral-coordinate S atoms (*μ*_4_-S) (every *μ*_4_-S binds to four Au atoms, see Fig. 3l), indicating the uniqueness of Au–S bonding in the Au_60_S_6_(SCH_2_Ph)_36_ nanocluster. Herein, the six surprising *μ*_4_-S atoms should come from the thiol, which may undergo S–C bond cleavage under heating conditions during the ligand-exchange-induced structure transformation process^42^. A single Au_20_S_3_(SR)_18_ staple motif (branched at the site of the *μ*_4_-S atom) contains two -Au-SR-Au- and three -Au-SR-Au-SR-Au- units between the five terminal *μ*_2_-S atoms and three bridging *μ*_4_-S atoms, and one -Au-SR-Au-SR-Au- and one -Au-SR-Au-SR-Au-SR-Au- units among the three bridging *μ*_4_-S atoms (Fig. 3j). Of note, such a giant staple motif has not been found in thiolated gold nanoclusters, which is larger than the Au_8_(SR)_8_ staple motif in Au_20_(SR)_16_ nanoclusters^27^.

### Crystallographic arrangement

**Figure 4:**
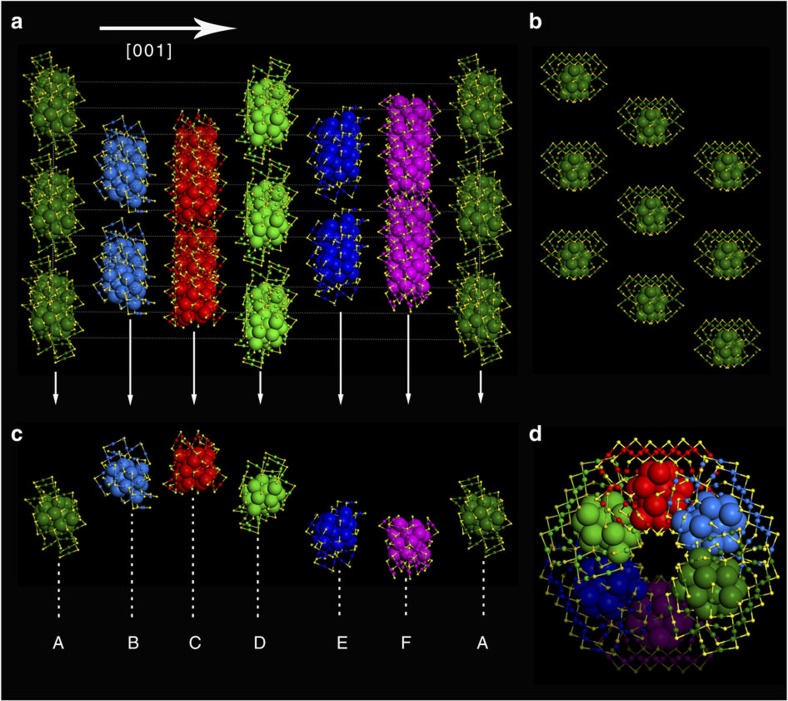
6HLH crystallographic arrangement of Au_60_S_6_(SCH_2_Ph)_36_ nanoclusters. (**a**) The stacking sequence along the [001] direction; (**b**) the closest-packed pattern of Au_60_S_6_(SCH_2_Ph)_36_ nanoclusters in the (001) plane; (**c**,**d**) different views of the left-handed helical arrangement of Au_60_S_6_(SCH_2_Ph)_36_ nanoclusters. (Note: to highlight the 6HLH arrangement, the Au atoms of the nanoclusters in each close-packed plane are labeled in different colours).

Interestingly, Au_60_S_6_(SCH_2_Ph)_36_ nanoclusters adopt the closest packing, and a very special stacking sequence of ‘ABCDEF' along the close-packed [001] direction is found in its single crystals (Fig. 4a). The Au_60_S_6_(SCH_2_Ph)_36_ nanoclusters in every stacking layer ((001) plane) are arranged uniformly, and each nanocluster is surrounded by six identical nanoclusters with the same tropism (**k**-vector), as shown in Fig. 4b and Supplementary Fig. 3. Moreover, the stacking layer perpendicular to the [001] direction can overlap completely with its neighboring layer after every nanocluster in the layer rotates (60°) clockwise or anti-clockwise along the *z* axis (Supplementary Fig. 3), and thus the arrangement of Au_60_S_6_(SCH_2_Ph)_36_ nanoclusters in single crystals along the [001] direction is reminiscent of the left-handed helix (Supplementary Fig. 4). For clarity, a left-handed helical sequence, here termed the 6H left-handed helical (6HLH) arrangement, is isolated from the crystal, as shown in Fig. 4c,d. Such a crystallographic arrangement is not only interesting but also exciting, as the third closest packing in crystals, named 4H, was found in 1979 (ref. 38).

### Photoluminescence

**Figure 5:**
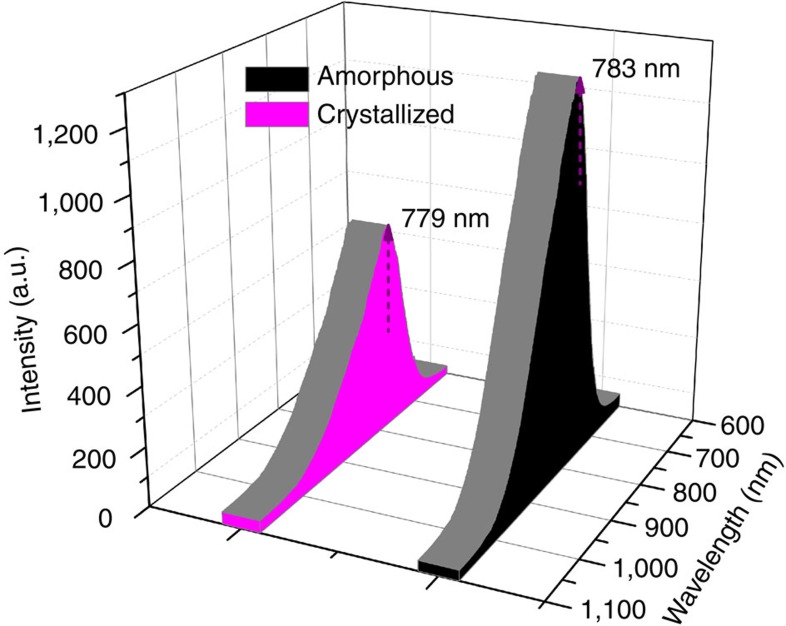
The solid photoluminescence spectra of Au_60_S_6_(SCH_2_Ph)_36_ nanoclusters. Au_60_S_6_(SCH_2_Ph)_36_ nanoclusters in crystallized and amorphous states exhibit obviously different photoluminescence intensities but almost identical emission spectrum profiles. Note: the excitation wavelength *λ*_ex_=514 nm.

**Figure 6:**
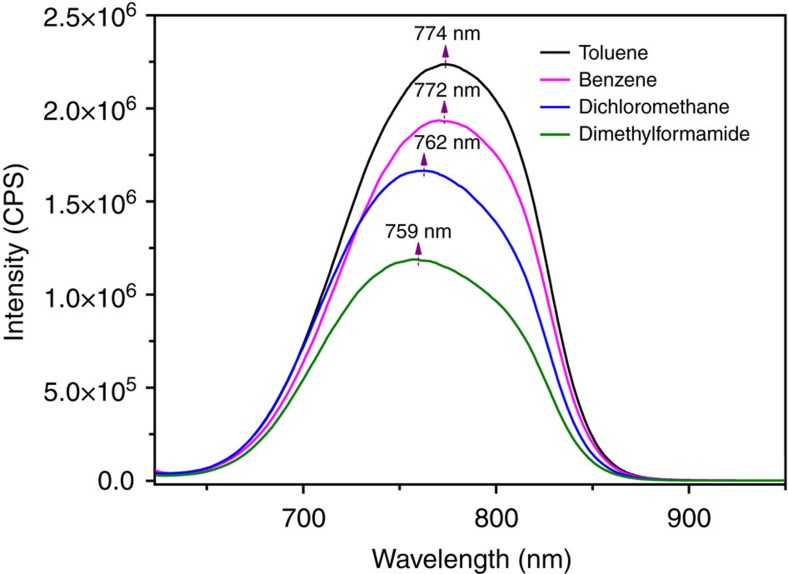
The solvent-polarity-dependent solution photoluminescence of Au_60_S_6_(SCH_2_Ph)_36_ nanoclusters. Not only the maximum emission wavelengths but also the photoluminescence intensities of Au_60_S_6_(SCH_2_Ph)_36_ nanoclusters in solution are dependent on the solvent polarity. Note: the excitation wavelength *λ*_ex_=514 nm.

The 6HLH arrangement indicates the unique interactions among Au_60_S_6_(SCH_2_Ph)_36_ nanoclusters in the single crystals, which is supported by the photoluminescence intensity comparison between the amorphous and crystallized Au_60_S_6_(SCH_2_Ph)_36_ as shown in Fig. 5. Although the emission spectrum profiles of the disordered (amorphous) Au_60_S_6_(SCH_2_Ph)_36_ is almost superimposable to that of the ordered (crystallized) Au_60_S_6_(SCH_2_Ph)_36_, the emission intensity of the former is ∼1.7 folds of that of the latter. The lower photoluminescence intensity in crystallized Au_60_S_6_(SCH_2_Ph)_36_ might be caused by the energy transfer among the 6HLH arranged Au_60_S_6_(SCH_2_Ph)_36_ nanoclusters, indicating the notable interactions among the 6HLH arranged Au_60_S_6_(SCH_2_Ph)_36_ nanoclusters. Although the 6HLH arrangement barely changes the emission spectrum, solvent does result in the obvious blue-shift of the maximum emission peak, and interestingly, the blue-shift increases with the increase of solvent polarity, while the photoluminescence intensity of Au_60_S_6_(SCH_2_Ph)_36_ nanoclusters decreases with the increase of solvent polarity (see Fig. 6). Anyway, these facts indicate that the solution photoluminescence of Au_60_S_6_(SCH_2_Ph)_36_ is solvent-polarity dependent.

## Discussion

In summary, a novel near-infrared-emissive gold nanocluster was synthesized via a thermal-induced ligand exchange process, and its composition was determined to be Au_60_S_6_(SCH_2_Ph)_36_ using ESI-MS and SCXC. SCXC also revealed that the nanocluster consists of one fcc-like Au_20_ kernel protected by a pair of Au_20_S_3_(SCH_2_Ph)_18_ giant staple motifs, which are larger than the existing staple motifs in structurally resolved gold nanoclusters. In particular, three tetrahedral-coordinate *μ*_4_-S atoms were found enclosed in every giant staple motif, which challenges the conventional opinion that sulfur in the Au–S interface is always three-coordinate *μ*_2_-S. Most important of all, a 6HLH crystallographic arrangement was found in the crystal of Au_60_S_6_(SCH_2_Ph)_36_ nanoclusters, and this finding also represents an advance in crystallographic packing research since the 4H phase finding in 1979. Interestingly, the 6HLH arrangement gives rise to the obvious loss of solid photoluminescence of amorphous Au_60_S_6_(SCH_2_Ph)_36_, indicating the strong interaction among the uniquely arranged nanoclusters. Another interesting finding is the solvent-polarity-dependent solution photoluminescence of Au_60_S_6_(SCH_2_Ph)_36_ nanoclusters. Briefly, this work provides new and exciting views to the structure, Au–S bonding and photoluminescence (including solid state and solution) of gold nanoclusters, and our work is expected to stimulate further research on the structure and properties of crystallized materials at the nanoscale.

## Methods

### Reagents

All chemicals are commercially available and used as received. Tetraoctylammonium bromide (TOAB, 98.0%), 2-Phenylethanethiol (PhC_2_H_4_SH, 99.0%) and phenylmethanethiol (PhCH_2_SH, 99.0%) were purchased from Sigma-Aldrich. Tetrachloroauric(III) acid (HAuCl_4_·4H_2_O, 99.7%), sodium borohydride (NaBH_4_, 98.0%), acetonitrile (99.0%, AR), dichloromethane (CH_2_Cl_2_, 99.0%, AR), tetrahydrofuran (THF, 99.0%, AR), toluene (99.5%, AR), methanol (CH_3_OH, 99.5%, AR), and petroleum ether (AR) were purchased from Sinopharm Chemical Reagent Co., Ltd.

### Synthesis of Au_60_S_6_(SCH_2_Ph)_36_ nanoclusters

All chemicals and reagents were used as received. Ten milligrams of Au_38_(SC_2_H_4_Ph)_24_ nanoclusters was dissolved in 1 ml of toluene containing 0.5 ml of PhCH_2_SH. Next, the reaction proceeded overnight at 100 °C under nitrogen atmosphere, and then was terminated by the addition of excess methanol. The crude product was washed with petroleum ether and methanol four times, dissolved in dichloromethane (DCM), and then subjected to separation and purification by PTLC. Single crystals of the purified nanoclusters were grown by the vapour diffusion of acetonitrile into a toluene solution of the purified nanoclusters at 5 °C, and black-coloured crystals formed after one week. Au_38_(SC_2_H_4_Ph)_24_ were prepared according to previous reports^44^.

### Characterization

Ultraviolet-visible-near-infrared absorption measurements were performed on a Shimadzu UV-3600 spectrophotometer (DCM as solvent). The single crystal diffraction data of Au_60_S_6_(SCH_2_Ph)_36_ was recorded on a Bruker APEXDUO X-ray Diffractometer (Bruker, Germany). ESI-MS was conducted on a Waters Q-TOF mass spectrometer equipped with a Z-spray source, and the source temperature was kept at 70 °C. To prepare the samples for ESI-MS analysis, Au_60_S_6_(SCH_2_Ph)_36_ was dissolved in toluene (∼0.5 mg ml^−1^) and then diluted (1/1, v/v) with an ethanol solution containing 0.5 mM CsOAc. The sample was directly infused into the chamber at 5 μl min^−1^. The spray voltage was 2.20 kV, and the cone voltage was kept at 60 V. The solution photoluminescence spectra of Au_60_S_6_(SCH_2_Ph)_36_ nanoclusters were recorded on a Fluorolog-3-21, Tempro-01 spectrofluorometer (HORIBA Jobin Yvon), and the excitation wavelength was kept at 514 nm with a slit of 10 nm (OD_514_∼0.047, measured by Ultraviolet-visible-near-infrared spectrophotometer). The solid photoluminescence spectra of Au_60_S_6_(SCH_2_Ph)_36_ nanoclusters were recorded on a laser confocal scanning Raman/fluorescence scope (HORIBA Jobin Yvon) and laser (514 nm) power is 0.5 mW. The PTLC plates were eluted with DCM/petroleum ether mixture (1/1, v/v) at room temperature under air atmosphere.

### Data availability

The X-ray crystallographic coordinates for structures reported in this article (see Supplementary Table 1 and Supplementary Data 1) have been deposited at the Cambridge Crystallographic Data Centre (CCDC), under deposition number CCDC 1526120. The data can be obtained free of charge from the Cambridge Crystallographic Data Centre via www.ccdc.cam.ac.uk/data_request/cif. All other data are available from the authors on reasonable request.

## Additional information

**How to cite this article:** Gan, Z. *et al*. The fourth crystallographic closest packing unveiled in the gold nanocluster crystal. *Nat. Commun.* **8,** 14739 doi: 10.1038/ncomms14739 (2017).

**Publisher's note**: Springer Nature remains neutral with regard to jurisdictional claims in published maps and institutional affiliations.

## Supplementary Material

Supplementary Information Supplementary Figures and Supplementary Table.

Supplementary Data 1 Crystal data of Au_60_S_6_(SCH_2_Ph)_36_ nanoclusters
