# Supplementary material for: The fourth crystallographic closest packing unveiled in the gold nanocluster crystal
Source: Nat Commun. 2017 Mar 24;8:14739. doi: 10.1038/ncomms14739 (PMC5376665; doi:10.1038/ncomms14739)
Supplement: Supplementary Information — Supplementary Figures and Supplementary Table. [file ncomms14739-s1.pdf]

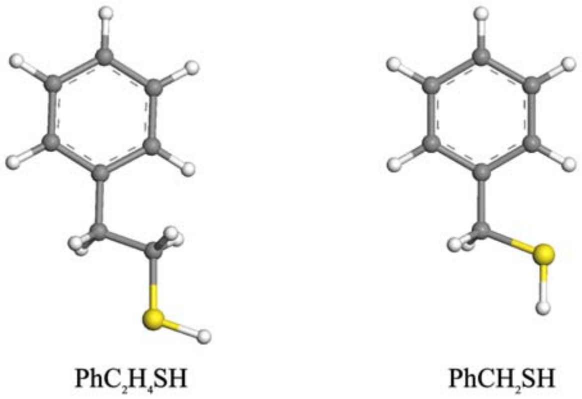

Supplementary Figure 1. The composition comparison between the protecting ligand of the starting nanoclusters and the incoming ligand. Color labels: yellow = S, gray = C, White=H.

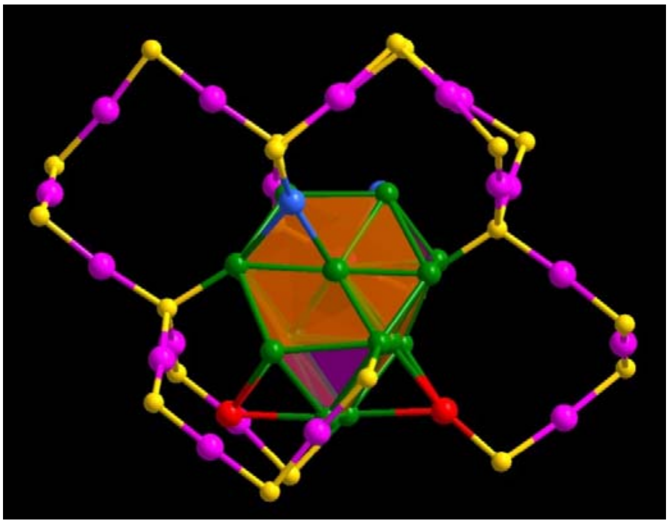

Supplementary Figure 2. Back view of Au_{20} kernel with a $Au_{20}S_3(SCH_2Ph)_{18}$ staple motif. Color labels: yellow = S, other color = Au.

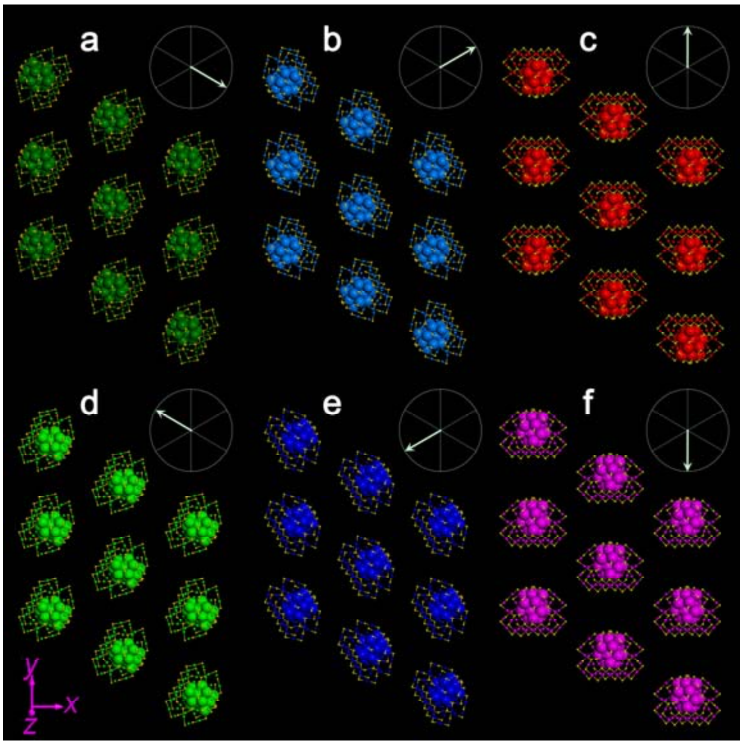

Supplementary Figure 3. (a-f) The arrangement and tropism of $Au_{60}S_6(SCH_2Ph)_{36}$ nanoclusters in the ABCDEF-sequenced layers ((001) planes). Note: to highlight the tropism, the Au atoms of the nanoclusters in each layer are labeled in different colors.

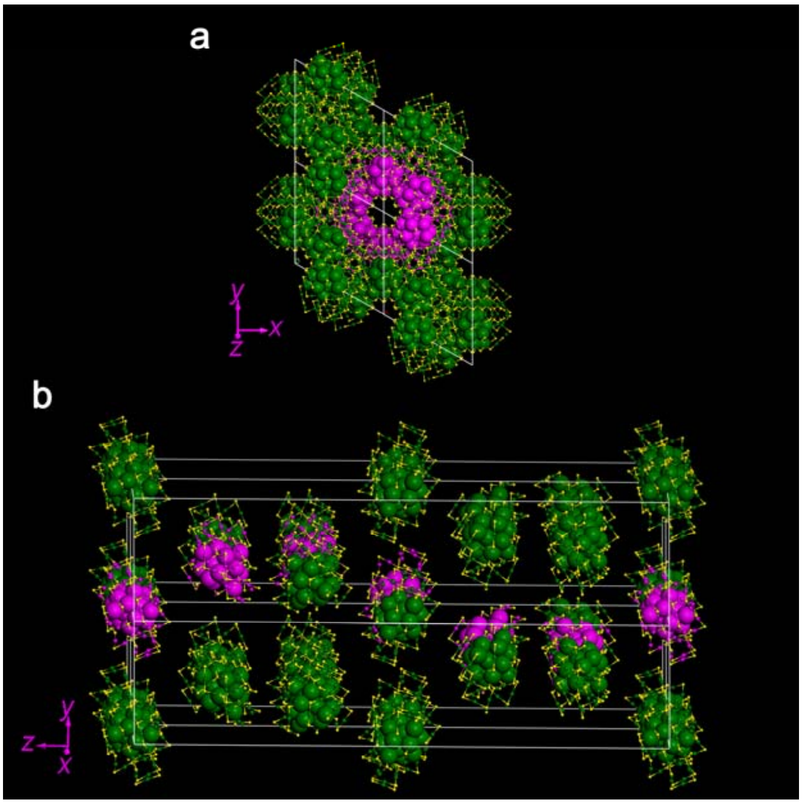

Supplementary Figure 4. The left-handed helical arrangement unit of $Au_{60}S_6(SCH_2Ph)_{36}$ nanoclusters in single crystals viewed in the direction parallel to the *z*-axis (a) or perpendicular to the *z*-axis (b).

Supplementary Table 1. Crystal data and structure refinement for $Au_{60}S_6(SCH_2Ph)_{36}$.

Identification code	$Au_{60}S_6(SCH_2Ph)_{36}$	
Empirical formula	$C_{252}H_{252}Au_{60}S_{42}$	
Formula weight	16445.03	
Temperature	173.0 K	
Wavelength	1.54178 Å	
Crystal system	Hexagonal	
Space group	*P*6522	
Unit cell dimensions	a = 23.7062(5) Å	a= 90°.
	b = 23.7062(5) Å	b= 90°.
	c = 95.566(3) Å	g = 120°.
Volume	46511(2) $Å^3$	
Z	6	
Density (calculated)	3.523 Mg/m^3	
Absorption coefficient	54.768 mm^{-1}	
F(000)	43056	
Theta range for data collection	2.152 to 68.272°.	
Index ranges	-28<=h<=28, -26<=k<=27, -114<=l<=113	
Reflections collected	267894	
Independent reflections	28219 [R(int) = 0.1652]	
Completeness to theta = 67.679°	99.4 %	
Absorption correction	Semi-empirical from equivalents	
Max. and min. transmission	0.1665 and 0.0206	
Refinement method	Full-matrix-block least-squares on F^2	
Data / restraints / parameters	28219 / 1779 / 1379	
Goodness-of-fit on F^2	1.139	
Final R indices [I>2sigma(I)]	R1 = 0.1027, wR2 = 0.2794	
R indices (all data)	R1 = 0.1684, wR2 = 0.3130	
Absolute structure parameter	0.15(6)	
Extinction coefficient	n/a	
Largest diff. peak and hole	4.947 and -2.870 e. $Å^{-3}$	
